# Urinary cotinine in tobacco farmers in Southern Brazil

**DOI:** 10.11606/S1518-8787.2018052000287

**Published:** 2018-07-13

**Authors:** Anaclaudia Gastal Fassa, Rodrigo Dalke Meucci, Nadia Spada Fiori, Maria Laura Vidal Carrett, Neice Muller Xavier Faria

**Affiliations:** I Universidade Federal de Pelotas. Departamento de Medicina Social. Programa de Pós-Graduação em Epidemiologia. Pelotas, RS, Brasil; II Fundação Universidade Federal de Rio Grande. Faculdade de Medicina. Programa de Pós-Graduação em Saúde Pública. Rio Grande, RS, Brasil; III Secretaria de Saúde de Bento Gonçalves. Vigilância em Saúde Ocupacional. Bento Gonçalves, RS, Brasil

**Keywords:** Farmers, Cotinine, urine, Tobacco, adverse effects, Sectional Studies

## Abstract

**OBJECTIVE:**

To describe urinary cotinine levels in tobacco farmers.

**METHODS:**

A cross-sectional study was conducted in 2,570 tobacco farmers. All participants that reported green tobacco sickness in the week prior to the interview plus a subsample of 492 pesticide applicators were included. We collected urinary samples and information about sociodemographic, behavioral, dietary, occupational characteristics, and pesticide poisoning during their lifetime. Stratification by sex and smoking was performed and the Wilcoxon and Kruskal-Wallis non-parametrical tests were used to analyze cotinine means.

**RESULTS:**

This study included 582 individuals. There was no difference in urinary cotinine means between green tobacco sickness symptomatic and asymptomatic individuals. Among non-smokers, having picked tobacco in the previous week was associated with higher cotinine means in both genders. Cotinine levels were higher on the first day of symptoms and reduced exponentially with each day in female non-smokers. Male non-smokers had higher levels on the second day and a more gradual reduction. The cotinine level rose up to 15 cigarettes/day of consumption.

**CONCLUSIONS:**

The urinary cotinine measures exposure to nicotine up to its saturation point; while green tobacco sickness, affected by tolerance, indicates nicotine poisoning. Strategies to reduce nicotine exposure in tobacco production are needed. Mechanization could be an alternative, as long as it overcame the challenge of irregular terrain and did not affect the quality of the leaf. More studies are needed to evaluate the chronic effect of nicotine exposure.

## INTRODUCTION

Cotinine is the main metabolite of nicotine and is an important marker of exposure to tobacco, whereby individuals with higher concentrations of cotinine are considered exposed to nicotine^5^. Among tobacco farmers, high levels of cotinine have been related to green tobacco sickness (GTS)^1^ ^,^ ^7^ ^,^ ^8^, which is an acute nicotine poisoning that happens mainly when tobacco leaves are harvested.

However, individuals constantly exposed to nicotine develop tolerance^10^. Smoking, along with other biological and behavioral factors, interferes with nicotine absorption by the skin and nicotine metabolism^3^. Therefore, the diagnosis of GTS is imprecise^1^ ^,^ ^6^, and its relationship with cotinine may vary in certain circumstances. The best moment to collect the cotinine depends on its pick in body fluids, but this has not been determined for dermic absorption yet. These aspects need to be considered when conducting epidemiological studies.

The objective of this article is to describe urinary cotinine levels in a sample of tobacco farmers, as well as to compare cotinine means in tobacco farmers with and without GTS symptoms in the previous week.

## METHODS

A cross-sectional study was carried out on tobacco farmers, in the state of Rio Grande do Sul, Brazil. To select a representative sample of 2,570 tobacco farmers, 1,100 tobacco sale invoices were randomly drawn from the 3,852 invoices issued, in the studied municipality, during the 2009 harvest. All individuals living in the properties of the selected invoices were interviewed. This study included 582 tobacco farmers, from the representative sample, from whom urinary cotinine was measured. The cotinine was measured in 492 tobacco farmers, with or without GTS symptoms, who applied pesticides in the year prior to the interview and who worked in properties located in the two districts with the highest tobacco production in the municipality; and in all 90 tobacco farmers from the representative sample who reported having symptoms of GTS. Data collection took place in the tobacco-harvesting period between January and March 2011.

The GTS was characterized by the following question: “In the last week, have you had or are you still having dizziness or headache, together with nausea or vomiting after picking tobacco?”. Urine samples were collected in vials, which were stored in a freezer at a temperature below -10ºC and sent weekly to the Toxicology Laboratory of the Pontifícia Universidade Católica do Rio Grande do Sul. Urinary cotinine was measured by chromatography using an ultraviolet detector.

A questionnaire was also administered to access sociodemographic information (gender, age, volume of tobacco production), behavioral and dietary characteristics (smoking and body mass index [BMI]), occupational characteristics (picking wet leaves, picking tobacco in the last week), symptoms after harvesting tobacco, and history of pesticide poisoning during lifetime.

The statistical chi-squared heterogeneity test was used to analyze differences in proportions between symptomatic and asymptomatic individuals. The differences between the genders on symptoms reported after harvesting tobacco were determined by Fisher’s exact test. Urinary cotinine means and medians were calculated, whereby stratification by gender and smoking was performed, excluding the small number of female smokers, in order to compare cotinine means according to the independent variables. Wilcoxon and Kruskal-Wallis non-parametrical tests were used. We also compared urinary cotinine means, according to the number of days of GTS symptoms prior to the urine sample being collected.

This study was approved by the Universidade Federal de Pelotas Research Ethics Committee (Official Letter 11/10, Record 40600038) and all the participants signed two informed consent forms, one to be interviewed and the other for urine to be collected.

## RESULTS

From the sample, 5.9% of the subjects were lost or refused to participate. In total, 582 individuals were interviewed and had urine samples collected; 137 were symptomatic and 445 were asymptomatic. Regarding the symptomatic individuals, 58.4% were female, 53.3% were aged 15–39, 14.6% were smokers, 87.6% picked wet leaves, and 96.3% had picked tobacco in the previous week. Regarding the asymptomatic individuals, 22.5% were female, 53.3% were aged 15–39, 22.7% were smokers, 81.6% picked wet leaves, and 91.2% had picked tobacco in the previous week (Table 1).

**Table 1:** Description of the tobacco farmers sample stratified by green tobacco sickness symptoms in the week preceding the interview. Brazil, 2011. (n = 582)

Variable	Symptomatic		Asymptomatic		p^a^
	
	n	%	n	%	
Gender					< 0.001
Male	57	41.6	345	77.5	
Female	80	58.4	100	22.5	
Age (years)					0.773
15–29	38	27.7	118	26.5	
30–39	35	25.6	119	26.8	
40–49	31	22.6	86	19.3	
≥ 50	33	24.1	122	27.4	
Tobacco production (kg)					0.003
1–5,000	56	40.9	118	26.5	
5,001–10,000	56	40.9	199	44.9	
10,001–36,000	25	18.2	126	28.4	
Smoking					0.121
No	117	85.4	344	77.3	
1–9 cigarettes /day	6	4.4	26	5.8	
≥ 10 cigarettes /day	14	10.2	75	16.9	
Secondhand smoke					0.550
No	79	57.7	270	60.7	
Yes	58	42.3	175	39.3	
Wet leaves harvest					0.119
No	17	12.4	82	18.4	
Yes	120	87.6	363	81.6	
Harvested in the last week					0.063
No	5	3.7	39	8.8	
Yes	132	96.3	406	91.2	
Pesticide poisoning during the lifetime					0.013
No	116	84.7	410	92.1	
Yes	21	15.3	35	7.9	
Body Mass Index^b^					0.310
Normal weight	15	45.5	213	48.0	
Overweight	11	33.3	177	39.9	
Obesity	7	21.2	54	12.2	

^a^ Fisher’s exact test of heterogeneity.

^b^ 464 individuals.

The GTS was defined as dizziness or headache symptoms, together with nausea or vomiting after picking tobacco. However, tobacco farmers reported other symptoms after harvesting tobacco, at some time in life. More than 9% of the males and 20% of the females mentioned weakness, sweating, insomnia, anorexia, agitation, and palpitation and females had significantly higher prevalence of all symptoms than males (Table 2).

**Table 2:** Distribution of the symptoms referred after harvesting tobacco, at some time in life, according to sex. Brazil, 2011. (n = 582)

Symptom	Male	Female	p*
	
	n (%)	n (%)	
Weakness			< 0.001
No	269 (66.9)	76 (42.2)	
Yes	133 (33.1)	104 (57.8)	
Sweating			0.019
No	292 (72.6)	113 (62.8)	
Yes	110 (27.4)	67 (37.2)	
Headache			< 0.001
No	324 (80.6)	104 (57.8)	
Yes	78 (19.4)	76 (42.2)	
Dizziness			< 0.001
No	343 (85.3)	110 (61.1)	
Yes	59 (14.7)	70 (38.9)	
Insomnia			< 0.001
No	344 (85.6)	127 (70.6)	
Yes	58 (14.4)	53 (29.4)	
Anorexia			< 0.001
No	349 (86.8)	132 (73.3)	
Yes	53 (13.2)	48 (26.7)	
Nausea			< 0.001
No	353 (87.8)	122 (67.8)	
Yes	49 (12.2)	58 (32.2)	
Agitation			< 0.001
No	355 (88.3)	131 (72.8)	
Yes	47 (11.7)	49 (27.2)	
Palpitation			0.001
No	363 (90.3)	144 (80.0)	
Yes	39 (9.7)	36 (20.0)	
Vomiting			< 0.001
No	364 (90.5)	140 (77.8)	
Yes	38 (9.4)	40 (22.2)	
Abdominal pain			0.017
No	363 (90.5)	150 (83.3)	
Yes	38 (9.4)	30 (16.7)	
Pallor			0.017
No	364 (90.5)	150 (83.3)	
Yes	38 (9.4)	30 (16.7)	
Dyspnea			< 0.001
No	379 (94.3)	151 (83.9)	
Yes	23 (5.7)	29 (16.1)	

* Fisher’s exact test.

Analysis stratified by gender and smoking did not show significant differences in urinary cotinine means when comparing symptomatic and asymptomatic individuals. Analysis of length of time with symptoms showed that female non-smokers with up to two days of symptoms had significantly higher cotinine means than those who had had symptoms for 3–5 and 6–7 days. Among males, either smokers or non-smokers, this relationship was not significant (p = 0.08 and 0.09, respectively) (Table 3).

**Table 3:** Mean and median (ng/ml) of urinary cotinine among tobacco farmers, according to smoking habit and gender. Brazil, 2011.

Variable	Female				Male							
	
	Non-smokers				Non-smokers				Smokers			
		
	n	Mean (SD)	Median	p	n	Mean (SD)	Median	p	n	Mean (SD)	Median	p
GTS symptoms in the previous week				0.715				0.660				0.370^a^
Symptomatic	76	158.7 (219.8)	84.5		41	181.5 (255.2)	115.0		16	789.6 (501.1)	701.2	
Asymptomatic	100	134.1 (154.6)	75.9		244	154.8 (209.6)	99.3		101	986.7 (688.6)	955.9	
Time of symptoms (days)^c^				< 0.001^b^				0.088^b^				0.088^b^
0–2	45	225.4 (260.0)	138.9		21	221.8 (328.0)	126.5		10	811.6 (482.8)	740.5	
3–5	24	75.3 (76.7)	55.9		14	174.0 (154.5)	138.5		4	421.6 (328.2)	434.6	
6–7	7	16.0 (21.8)	0.0		6	58.0 (57.5)	38.2		2	1415.7 (121.1)	1415.7	
Cigarettes/day				-				-				< 0.001^b^
1–5	-	-	-		-	-	-		21	454.1 (504.7)	257.5	
6–10	-	-	-		-	-	-		35	1074.0 (718.0)	957.4	
11–15	-	-	-		-	-	-		20	1356.6 (560.6)	1387.6	
16–20	-	-	-		-	-	-		25	798.3 (514.0)	771.1	
≥ 21	-	-	-		-	-	-		16	1129.5 (668.2)	1365.5	

GTS: green tobacco sickness

^a^ Wilcoxon test for comparison of means.

^b^ Kruskall-Wallis test for comparison of means.

^c^ Only symptomatic individuals.

The mean level of urinary cotinine continued to rise until the consumption of 15 cigarettes a day was reached by male smokers. Consumption above this amount did not result in increased cotinine levels (Table 3).

Among female non-smokers, cotinine levels were higher on the first day of symptoms and reduced exponentially with each successive day. Male non-smokers had higher levels on the second day and a more gradual reduction than females (Figure).

**Figure:**
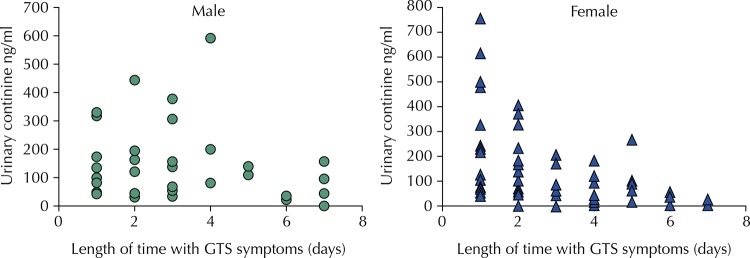
Urinary cotinine distribution according to length of time with GTS symptoms (days) among non-smokers.

GTS: green tobacco sickness

Among non-smokers, having picked tobacco in the previous week was associated with higher cotinine means in both genders. Older women had lower urinary cotinine means in relation to younger women and women who picked wet tobacco leaves had higher means. Among men, the amount of tobacco production was positively associated with cotinine levels. In the case of male smokers, only age was positively associated with urinary cotinine means. BMI and pesticide poisoning were not associated with cotinine means in the analysis stratified by gender and smoking habit (Table 4).

**Table 4:** Mean and median (ng/ml) of urinary cotinine according to independent variables, stratified by gender and smoking habit. Brazil, 2011.

Variable	Female				Male							
	
	Non-smokers				Non-smokers				Smokers			
		
	n	Mean (SD)	Median	p	n	Mean (SD)	Median	p	n	Mean (SD)	Median	p
Cotinine	176	144.7 (185.4)	79.3		285	158.7 (216.4)	99.5		117	959.7 (667.7)	947.2	
Age				0.014^a^				0.132^a^				0.040^a^
15–29	50	177.9 (174.6)	132.5		89	192.8 (265.4)	123.9		17	559.4 (477.4)	293.6	
30–39	41	129.1 (162.2)	73.0		85	158.3 (227.6)	89.9		27	1083.4 (672.4)	957.4	
40–49	41	194.0 (265.4)	85.6		47	143.1 (206.8)	79.8		27	957.5 (630.9)	782.8	
≥ 50	44	75.7 (80.4)	52.5		64	123.2 (96.8)	96.1		46	1036.4 (708.1)	1159.1	
Tobacco Production (kg)				0.057^a^				< 0.001^a^				0.430^a^
1–5,000	53	137.4 (242.1)	52.5		79	126.8 (195.1)	59.3		40	1035.2 (640.0)	996.7	
5,001–10,000	84	142.5 (157.6)	95.9		124	125.0 (112.8)	98.6		46	927.5 (673.8)	1064.5	
10,001–36,000	39	159.5 (153.3)	99.8		81	241.8 (316.0)	140.1		30	896.9 (714.4)	748.7	
Secondhand smoke				0.944^b^				0.237^b^				0.323^b^
No	81	134.1 (152.0)	82.6		202	150.6 (197.0)	95.3		64	883.3 (597.3)	777.0	
Yes	95	153.8 (210.1)	76.0		83	178.4 (258.0)	119.5		53	1052.0 (739.3)	1070.4	
Wet leaves harvest				0.028^b^				0.089^b^				0.134^b^
No	26	88.1 (143.0)	45.9		52	116.7 (132.9)	74.0		20	809.6 (844.9)	421.7	
Yes	150	154.6 (190.5)	92.6		233	168.0 (230.2)	108.9		97	990.7 (625.9)	985.4	
Harvested in the last week				0.001^b^				< 0.001^b^				0.476^b^
No	17	57.9 (106.2)	25.5		19	52.2 (80.4)	18.6		8	1102.9 (622.0)	1197.7	
Yes	159	154.0 (189.8)	91.1		266	166.3 (221.1)	111.3		109	949.2 (670.0)	918.3	
Pesticide poisoning during the lifetime				0.120^b^				0.104^b^				0.352^b^
No	161	151.6 (191.3)	85.64		254	165.1 (225.4)	105.2		108	947.0 (672.2)	930.4	
Yes	15	70.9 (72.2)	50.66		31	106.1 (109.4)	80.2		9	1111.9 (626.4)	1330.1	
Body Mass Index^c^				0.913^a^				0.760^b^				0.473^a^
Normal weight	35	142,9 (158.7)	104.9		129	143.3 (164.7)	99.5		56	1000.3 (716.8)	956.7	
Overweight	55	125.9 (153.7)	66.4		95	153.3 (174.7)	98.9		35	1028.4 (638.4)	1073.5	
Obesity	22	114.2 (111.4)	83.5		27	200.3 (403.9)	80.9		10	729.0 (649.2)	454.6	

^a^ Kruskall Wallis test for comparison of means.

^b^ Wilcoxon test for comparison of means.

^c^ 464 individuals.

## DISCUSSION

Gender and smoking influence urinary cotinine levels in population studies and require stratification^3^. Urinary cotinine means were much higher in smokers than in non-smokers. There was no difference in cotinine means between the genders among non-smokers, although cotinine levels did behave differently depending on the length of time of the symptoms. Females had a cotinine peak before males and a more accelerated reduction over time, thus reinforcing the differences in cotinine metabolism and excretion between males and females described in other studies in which females metabolized and excreted nicotine quicker than males^3^.

The GTS criteria used in this study was based on several international studies^1^; however, it has low specificity since the criteria are based on symptoms common to other morbidities. Among the symptoms frequently reported by tobacco farmers, weakness and sweating might be related to the strenuous work while harvesting in hot days. However, insomnia, anorexia, agitation, and palpitation could be related to nicotine exposure and its higher frequency in females could be explained in part by cultural reasons, as they perceive and report more symptoms than men, and also by their higher susceptibility to nicotine. Studies have reported other symptoms after harvesting, suggesting the need to extend the approach of GTS^1^.

Consistent with other studies, females reported more GTS symptoms despite having cotinine means similar to males. Although women often report more symptoms than men, this finding reinforces the hypothesis that there are differences in the development of tolerance between the genders^6^ ^,^ ^7^. Studies have shown that individuals who are fast metabolizers of nicotine lose more rapid tolerance when compared to those who metabolize more slowly^3^ ^,^ ^9^. This would mean that women are more sensitive to the effects of nicotine than men.

The absence of a relationship between urinary cotinine means and the occurrence of GTS symptoms, regardless of gender and smoking habits, shows that urinary cotinine is not an indicator of nicotine poisoning but rather a marker of the intensity of exposure, whilst GTS indicates greater susceptibility to nicotine. The mechanism of tolerance to nicotine shows great individual variability and plays an important role in the occurrence of GTS. Symptoms of GTS are also common in heat stress and other forms of poisoning, including pesticides, and this may result in nondifferential classification errors of GTS, but pesticide poisoning was not associated with cotinine means.

Associated factors were different according to smoking habit. Among male smokers only age was relevant, higher cotinine means in older people may be related to a reduction in nicotine clearance in those aged over 65, as well as to a possible higher cigarette consumption^3^. Among non-smokers, occupational factors were also associated. Among female non-smokers, age may be a marker of the division of labor if older tobacco farmers reduce their participation in the harvest due to the high level of physical exertion required. The higher cotinine levels in female non-smokers who pick wet tobacco leaves is consistent with studies that indicate the role of water (moisture) in facilitating nicotine absorption^1^ ^,^ ^2^ ^,^ ^7^, given that it is soluble in both polar and non-polar substances.

The amount of tobacco produced acted as an indicator of nicotine exposure intensity among male non-smokers. A Korean study measured the concentration of nicotine present in the air both in harvesting fields and in drying barns and found levels several hundred times higher than those permitted for the workplace^11^, thus proving that workers are exposed to high concentrations of nicotine not only through the skin but also by breathing during tobacco drying and processing. In a workplace with high airborne nicotine concentrations, passive smoking did not determine significant levels of cotinine among non-smokers.

Among male smokers, smoking is the main factor responsible for high cotinine levels and suggests the existence of a saturation point for daily nicotine absorption, peculiar to each individual^4^, which could cause a reduction in nicotine absorption after reaching this point. In this study, urinary cotinine levels continued to rise until reaching the point of consumption of 15 cigarettes per day (possible saturation point) and stabilized following greater cigarette consumption. The saturation point found in the male tobacco farmers was lower than that found by Blackford et al.^4^ in a general population (20 cigarettes a day), which is probably related to the occupational exposure to nicotine in tobacco farming. The limitations of this study include the lack of multivariable analyses due to the asymmetry of the cotinine distribution, the lack of cotinine levels adjustment for urinary creatinine and the absence of information about factors that influence cotinine metabolism, such as race, genetic variation of the CYP2A6 enzyme, pregnancy, and liver and kidney disease.

In view of the direct relationship between smoking and cotinine levels and its role in the development of nicotine tolerance, future studies should emphasize the need to describe the amount and the type of tobacco consumed, in addition to measure environmental exposure to nicotine and control for factors that influence nicotine metabolism, ensuring that the sample size is sufficient to be stratified by gender and smoking. Studies with other designs are needed to investigate the chronic effects of high exposure to nicotine as well as whether differences in tolerance to such exposure are reflected in the long-term effects on health.

This paper subsidizes the discussions of the working groups on article 18 of the Framework Convention on Tobacco Control, which deals with the protection of the environment and human health. The high cotinine levels in tobacco farmers indicate the need to define strategies to reduce nicotine absorption, particularly during tobacco harvesting. The hot climate during the harvest limits personal protective equipment use, and, frequently, personal protective equipment used are not certified to prevent dermal absorption of nicotine. Harvest mechanization should be considered, but technology needs to be developed to deal with rough ground, typical of the studied region, and to guarantee the leaf quality. Policies addressing crop diversification are also important to promote farmer’s sustainability.
